# Electronically Driven Magnetoelectric Coupling in Co/La:Hf_0.5_Zr_0.5_O_2_ Heterostructures for Energy-Efficient Neuromorphic Computing

**DOI:** 10.1021/acsami.5c22784

**Published:** 2026-02-25

**Authors:** Alberto Quintana, Cesar Magen, Mehrdad Ghiasabadi Farahani, Wenjing Dong, Jingye Zou, Nico Dix, Zheng Ma, Enric Menéndez, Michael Foerster, Miguel Angel Niño, Claudio Cazorla, Jordi Sort, Florencio Sánchez, Ignasi Fina

**Affiliations:** † 54449 Institut de Ciència de Materials de Barcelona (ICMAB-CSIC), Campus UAB, Bellaterra, 08193 Barcelona, Spain; ‡ Instituto de Nanociencia y Materiales de Aragón (INMA), CSIC-Universidad de Zaragoza, 50009 Zaragoza, Spain; § Departamento de Física de la Materia Condensada, 16765 Universidad de Zaragoza, 50018 Zaragoza, Spain; ∥ Departament de Física, 16719 Universitat Autònoma de Barcelona, 08193 Cerdanyola del Vallès, Spain; ⊥ ALBA Synchrotron Light Source, CELLS, CCerdanyola del Vallès, E-08290 Barcelona, Spain; # Group of Characterization of Materials, Departament de Física, 16767 Universitat Politècnica de Catalunya, Campus Diagonal Besòs, Av. Eduard Maristany 10−14, 08019 Barcelona, Spain; ¶ Research Center in Multiscale Science and Engineering, Universitat Politècnica de Catalunya, Campus Diagonal-Besòs, Av. Eduard Maristany 10−14, 08019 Barcelona, Spain; ∇ Institució Catalana de Recerca i Estudis Avançats (ICREA), Pg. Lluís Companys 23, E-08010 Barcelona, Spain; ○ Catalan Institute of Nanoscience and Nanotechnology (ICN2), CSIC and BIST, Campus UAB, Bellaterra, 08193, Barcelona, Spain; 10 Instituto de Química Física Blas Cabrera, CSIC, Madrid E-28006, Spain

**Keywords:** ferroelectric, HfO_2_, magnetoelectric, HZO, neuromorphic computing

## Abstract

Magnetoelectric materials enable low-power memory devices by leveraging the electric control of magnetization. The discovery of ferroelectricity in doped hafnia has unlocked further opportunities since the distinct ferroelectric switching mechanism in this material can enable robust and multilevel modulation of magnetization by electric field, if combined with appropriate magnetic materials. Here, we demonstrate a 5% electric field-induced modulation of the saturation magnetization in a cobalt layer, driven by ferroelectric switching of an adjacent epitaxial La­(1%):Hf_0.5_Zr_0.5_O_2_ film. Dichroic imaging with synchrotron radiation confirms that ferroelectric switching induces a magnetic change. We show that the response time is faster than 500 ns (limited by the setup time resolution threshold) and that energy consumption is 6 nJ. This low energy consumption is mainly enabled by the absence of relevant leakage current contribution (10 nA/cm^2^ at 500 mV). The found response time and energy-efficient behavior point to the presence of an electronically driven modulation of magnetism (i.e., conventional magnetoelectric effects), which is confirmed by theoretical calculations and compositional analysis. Additionally, a multilevel magnetoelectric response is observed, enabling neuromorphic-like behavior. The demonstration of magnetoelectric coupling in a system based on CMOS-compatible materials offers a viable route toward the development of low-power beyond von-Neumann technologies.

## Graphical abstract

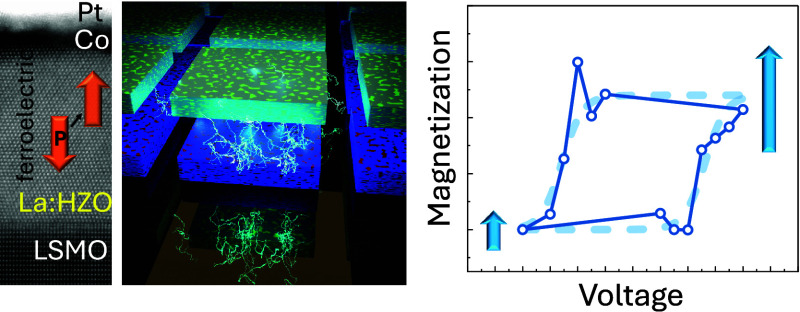

## Introduction

Memory technologies have importantly relied on the use of magnetic materials.^1^ This is primarily because magnetic order enables a significantly low standby energy consumption, allowing for efficient, long-term data storage. However, the manipulation of magnetic order requires the injection of large electrical currents, whether the switch of the magnetic order is achieved through the magnetic torque generated by a magnetic field or a spin-polarized current, both involving high energy consumption (≈100 fJ/bit in optimized devices).^2^ Undesired Joule heating, which occurs alongside the presence of electrical currents, and limitations regarding 3D scalability^3^ are other factors that can limit the use of magnetic materials in the post-von Neumann era.^2–5^ Eventually, the use of magnetoionic coupling (i.e., manipulation of magnetism through voltage-triggered ion migration) can potentially result in large changes in magnetization using electrolytes^4^ or ionic conductors.^5^ However, magnetoionic coupling requires the injection of electrical current though the device and its response time is, therefore, found to be consistently slow, typically around ≈10 ms or slower^6–8^ due to the inherently slow response time of the underlying ion diffusion and electrochemical reaction processes.

Transitioning from current-based approaches to electric-field-based magnetic writing holds great promise for the implementation of energy-efficient technologies.^9^ The development of multiferroic heterostructures, composites comprising adjacent ferroelectric and ferromagnetic materials, enables the coexistence of both ferroic orders above room temperature, resulting in deterministic electric-field control of magnetization.^10,11^ The mechanisms^12–14^ that trigger the coupling between electric fields and magnetization have been the subject of intensive research for many years. These include electric-field-driven modulation of carrier density in the magnetic layer^15–18^ and modulation of magnetic properties by strain coupling between piezoelectricity and magnetostriction of the ferroelectric and ferromagnetic materials, respectively.^15,19–21^ In multiferroic systems based on ferroelectrics such as BaTiO_3_,^22^ BiFeO_3_,^23,24^ and PVDF,^25,26^ changes in the coercive magnetic field or magnetic domain configuration under electric stimuli have been reported. However, none of these materials are compatible with CMOS technology. Additionally, a failure rate of ≈50% of devices was reported by Vaz et al. in systems based on BiFeO_3_/Co,^27^ further impeding device implementation. Larger effects can be found by leveraging the AFM to FM transition present in FeRh near and above room temperature.^28–32^ However, in this system, magnetoelectric coupling is mediated by large strain changes, thereby rendering its integration into devices particularly challenging due to clamping effects.^33^

The limited progress in developing magnetoelectric devices that are able to be integrated into computing architectures underscores the urgent need to investigate systems where distinct mechanisms may be at play. Ferroelectric doped hafnia is attracting great attention from the academic and industrial communities.^34^ Ferroelectric hafnia is CMOS compatible, making it possible to overcome the challenging integration of magnetoelectric composites based on perovskite ferroelectrics, and mechanisms that differ from those found in ferroelectric perovskites are present. Therefore, the study of magnetoelectric coupling in hafnia-based devices is extremely interesting from a fundamental and technological point of view.^35^ Large-anisotropic room-temperature ferromagnetism in hafnia arising from structural defects has been observed.^36^ However, the extrinsic nature of the multiferroic order in hafnia allows one to anticipate that magnetoelectric coupling should not be present. Therefore, the combination of ferroelectric HfO_2_ with a ferromagnetic material is a more promising option.

Surprisingly, investigations of magnetoelectric coupling in systems based on ferroelectric HfO_2_ are scarce. Changes in magnetization up to 25% in HfO_2_/Ni have been predicted.^37,38^ Experimentally, magnetoelectric coupling in HfO_2_/Ni heterostructures has been tested by X-ray absorption measurements.^39^ It was suggested that spontaneous redox processes at the metal–ferroelectric interface were relevant. The coexistence of both charge-driven and ionic modulation of Fe magnetic properties in Hf_0.5_Zr_0.5_O_2_/Fe heterostructures has been also proposed.^40^ Furthermore, evidence of changes in the magnetization in the CoPt/Al:HfO_2_ structure upon ferroelectric switching has been reported.^41^ In all of these cases, the strong reactivity of magnetic electrodes resulted in a severe degradation of ferroelectric properties. The chemical interaction is unavoidable when using polycrystalline HfO_2_ films, where crystallization by annealing is required. The insertion of a thin interlayer between the ferromagnetic and ferroelectric materials^42^ mitigates this undesired effect, but with an evident negative impact on the magnetoelectric coupling.

Epitaxial hafnia films show higher crystalline quality and absence of the wake-up effect.^43^ In addition, epitaxial films do not require postannealing and show smooth surfaces, in contrast to polycrystalline samples,^44^ allowing very sharp interfaces. As concluded by Dmitriyeva et al.,^39^ since preliminary charge-mediated magnetoelectric coupling effects have been observed in systems based on polycrystalline HfO_2_, it is expected that heteroepitaxial structures will exhibit a much stronger coupling. These systems are not CMOS-compatible, but their superior structural and morphological properties make them an appropriate platform to study intrinsic effects and potentially observe larger responses, providing insights needed to guide the design of future commercial devices. Indeed, the higher quality of epitaxial films leads to remarkable multiferroic properties with high polarization, endurance, retention, and sizable direct magnetoelectric effects.^45^ Polarization-driven changes in tunnel magnetoresistance driven by electric fields have also been reported.^46,47^ However, none of the previous studies has provided direct evidence of magnetization changes induced by ferroelectric switching nor have addressed key aspects such as characterization of response time and energy consumption.

Here, we demonstrate that sizable electrically induced changes in saturation magnetization up to 5% can be observed in a multiferroic system formed by top Co on a La(1%):Hf_0.5_Zr_0.5_O_2_ (La:HZO) film. These results are confirmed by X-ray magnetic circular dichroism (XMCD) in combination with photoemission electron microscopy (PEEM) measurements, where it is inferred that magnetic contrast also changes upon ferroelectric switching. The increase of magnetization for polarization state pointing away from the magnet (downward) and the response time (<500 ns) and low energy consumption (≈6 nJ) agree with conventional magnetoelectric effects, further confirmed by density functional theory (DFT) calculations. Finally, we harness the electric-field control of magnetization to demonstrate that magnetization can be modulated in a continuous manner (i.e., multilevel magnetoelectric response) by varying the pulse width (τ_w_) and amplitude (*V*_w_) of the electric stimuli.

## Results

### Magnetoelectric Switching

Figure 1a shows a schematic representation of the magnetoelectric system. An epitaxial La:HZO (15 nm) layer was grown on the La_0.67_Sr_0.33_MnO_3_ (LSMO, 25 nm) bottom electrode previously deposited onto a SrTiO_3_(001) substrate. Here, La doping of 1% was selected to obtain low leakage, while maintaining high polarization and endurance.^48^ Structural characterization shows that the orthorhombic phase of La:HZO is stabilized with a (111) out-of-plane orientation (see Figure S1 and also additional characterization of equivalent films reported elsewhere^48^). On top, an array of ≈200 square structures (60 × 60 μm^2^) of Pt (3 nm)-capped Co (1.5 nm) were grown by DC sputtering using a stencil mask and are shown in the inset of Figure 1a. These Pt/Co structures act simultaneously as top electrodes and active magnetic layers. Shown in Figure 1b are the current and voltage signals vs time for a representative electric voltage pulse of τ_w_ = 2.5 μs and a writing voltage of *V*_w_ = 8 V. Displayed (Figure 1b upper panel) current dependencies correspond to the switchable plus nonswitchable (*I*_SW_ + *I*_NSW_) and to nonswitchable (*I*_NSW_) responses. As in the Positive-Up-Negative-Down (PUND) technique,^49^ the switchable contribution [*I*_SW_ = (*I*_SW_ + *I*_NSW_) – (*I*_NSW_)] is extracted, which accounts for the switched ferroelectric polarization. From the direct inspection of the current peak, it can be inferred that its width is <500 ns. This value is not the intrinsic ferroelectric switching time since it is limited by the time constant of the measurement circuitry; thus, it represents an upper limit. Dedicated experiments have demonstrated ferroelectric switching within a few nanoseconds in both ferroelectric^50,51^ and multiferroic^52^ systems. More recently, switching times as short as 210 ps^53^ have been reported in HZO-based capacitors. These findings indicate that an ultrafast response is, in principle, achievable if the magnetoelectric response is driven by ferroelectric switching, as discussed below. The energy delivered to the system is calculated using the equation energy = ∫*I*·*V* d*t*, where *I* and *V* represent the time-dependent switching current and voltage, respectively. The resulting energy versus time curve is also shown in Figure 1b (bottom panel). The intrinsic energy consumption is about 3 nJ. In practice, nonswitchable contributions must also be considered, leading to a total energy consumption of approximately 6 nJ. It is remarkable that the current rapidly zeroes after the current peak occurs, indicating the negligible presence of leakage current through the device, which is further corroborated by the low leakage current below 10 nA/cm^2^ at 500 mV (see Figure S2). Considering that the device area is 3600 μm^2^ and thickness is 16.5 nm, i.e., the thickness of La:HZO (15 nm) plus Co (1.5 nm) layers, the energy density would result in 100 J/cm^3^, which corresponds to an areal density of 0.1 mJ/cm^2^. Scaling down the device dimensions is therefore expected to substantially reduce energy consumption.

**Figure 1:**
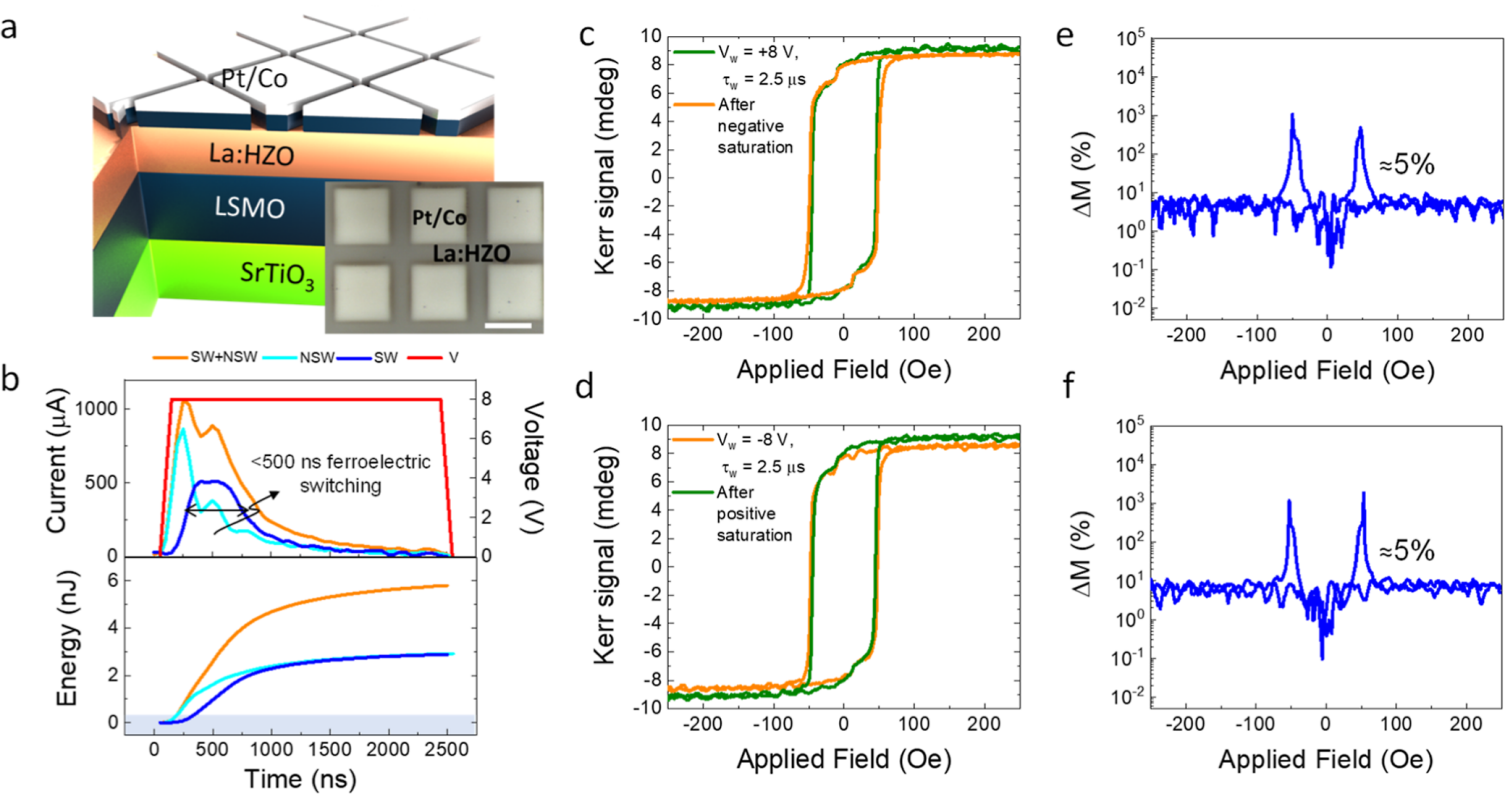
(a) Schematic of the Pt/Co/La:HZO/LSMO/SrTiO_3_ sample. Inset: top view of the Pt/Co on top of the La:HZO layer. Scale bar 50 μm. (b) Current, voltage, and energy versus time during the application of a voltage pulse of *V*_w_ = 8 V and τ_w_ = 2.5 μs. (c,d) Kerr magnetic loops after saturation and subsequent *V*_w_ = +8, −8 V voltage pulses of τ_w_ = 2.5 μs, respectively. (e,f) Δ*M* versus magnetic field extracted from (c,d), respectively.

The magnetoelectric response has been characterized by the magneto-optical Kerr effect (MOKE) with all magnetic measurements performed in-plane and at electrical remanence. While MOKE does not provide a quantitative determination of the absolute magnetization magnitude, the proportional relationship between Kerr rotation and magnetization allows the evaluation of relative magnetization changes under different ferroelectric poling states. Figure 1c,d displays the hysteresis loops of a characteristic magnetoelectric response of our Co/La:HZO multiferroic heterostructures. Both panels show the magnetic hysteresis loops at opposite ferroelectric remanent states obtained after saturation using a voltage pulse of opposite polarity long enough to ensure full ferroelectric saturation (τ_w_ = 2.5 ms, *V*_w_ = −8 and +8 V, for Figure 1c,d, respectively) compared to loops after a shorter inverted pulse is applied (τ_w_ = 2.5 μs, *V*_w_ = +8 and −8 V, for Figure 1c,d, respectively). For the latter short pulse, it can be observed that magnetic saturation increases for positive *V*_w_, i.e., for polarization pointing downward and away from Co. The opposite is observed for negative *V*_w_. Figure S3 shows that the total switched polarization is ≈15 μC/cm^2^. Figure S4 shows a replica experiment of that shown in Figure 1c,d, with a very small deviation, indicating the reliability of the 5% difference. In Figure 1e,f, the relative change of magnetization [Δ*M* = (*M*_H_ – *M*_L_)/*M*_L_, where *M*_H_ and *M*_L_ are the high and low Kerr signals from the data shown in Figure 1c,d, respectively], has been plotted as a function of magnetic field. It can be observed that at magnetic saturation the change is ≈5% in both cases. Near the coercive field, where magnetization is reduced, the relative ratio increases.

### Imaging of Magnetization Change under Electrical Stimuli

Prior to magnetic imaging, the overall magnetization is zeroed, i.e., the samples have been demagnetized. Demagnetization is done to better observe changes in the magnetic domain configuration, since after saturation fewer domains are present, making it more difficult to identify variations. Then several devices were electrically poled (*P*_up_ and *P*_down_) for synchrotron-based magnetic characterization and imaging. Figure 2a–c, shows XMCD-PEEM images acquired at 777 eV, corresponding to the L_3_ Co transition, acquired for the as-grown downward state (see Figure S5, where piezoelectric force microscopy (PFM) characterization is used to infer the as-grown state for ferroelectric polarization) and after the electrical poling (*V*_w_ = −8 and +8 V, with τ_w_ 2.5 μs, respectively), all at zero magnetic field. Similar images obtained in other devices of the sample are shown in Figure S6. It can be observed that the images appear brighter when the polarization points away from Co, which is, as a first approximation, consistent with the larger magnetization observed in MOKE experiments. However, it must be noted that image brightness is influenced not only by absolute magnetization but also by its orientation with respect to the beam and extrinsic effects, such as surface charging. The domains are mostly isotropic, which agrees with the 4 crystalline variants and 3 different possible directions for the ferroelectric polarization vector of La:HZO, resulting in an expected lack of anisotropy along in-plane directions (see Video S1).^54^ Possible variations of oxygen upon ferroelectric switching have not been detected by characterizing X-ray absorption near oxygen and cobalt edges, as shown in Figures S7 and S8, respectively. It is remarkable that the absence of variations of the oxidation state upon ferroelectric switching is observed in at least up to 10^3^ electrical cycles (Figure S9). Figure 2d shows a representative X-ray absorption spectroscopy (XAS) for circularly polarized clockwise (CW) and counterclockwise (CCW) light and XMCD spectra, extracted from a pristine device. The XMCD-PEEM image of the area where XAS and XMCD spectra were obtained is included as an inset. The XAS spectrum corresponds to that of bare Co, confirming that the signal is mainly contributed by metallic Co. The XMCD spectrum also corresponds to the archetypal shape of magnetic cobalt. Figure 2e shows the histograms of the XMCD intensity in the as-grown state, *P*_up_ and *P*_down_. The presence of a more pronounced double peak obtained in the as-grown state and after the application of +8 V, which accounts for the XMCD contrast, is directly proportional to the magnetization projected along the X-ray incidence. This indicates that a larger magnetization is present in these two states compared with the one obtained after the application of −8 V as summarized in the XMCD contrast plot of Figure 2f and in agreement with the MOKE experiments. Note that the contrast is larger after +8 V than in the as-grown state, likely due to the presence of some residual *P*_up_ domains in the as-grown state that result in a decrease in magnetization decrease.

**Figure 2:**
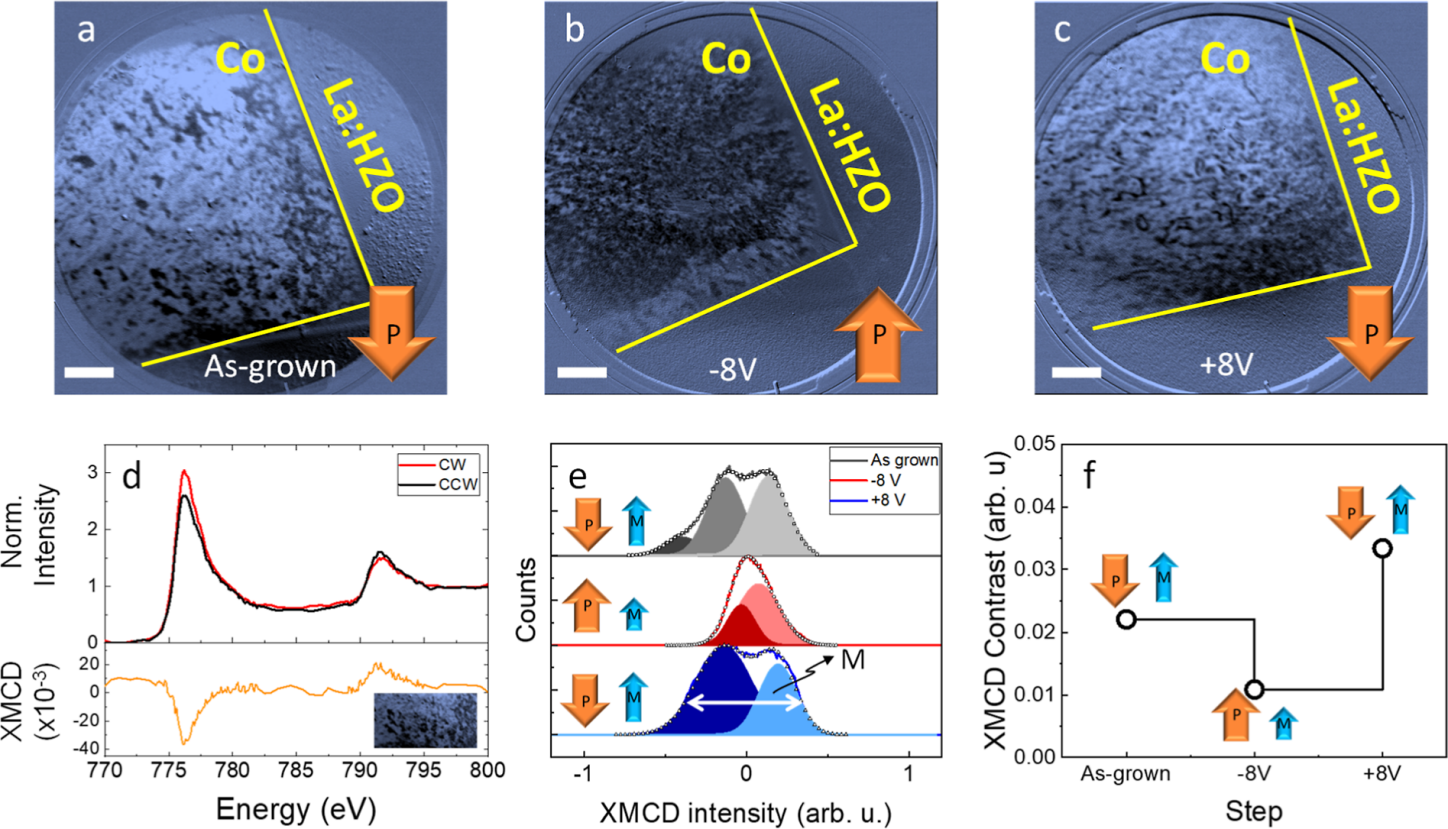
XMCD-PEEM images at the corner of a device where Co and La:HZO surfaces are visible. In (a) in the as-grown state and in (b,c) after application of *V*_w_ = −8 V and *V*_w_ = +8 V of 2.5 μs, respectively. Scale bars correspond to 5 μm. (d) XAS and XMCD spectra of the region shown in the inset. (e) Histograms of the XMCD intensity of the images shown in (a–c). (f) XMCD contrast at the indicated states.

### Conventional Magnetoelectric Effect

As shown in Figures 1 and 2, we only observe minor changes in the shape of the magnetic hysteresis loops and no change of the irregular shape of the magnetic domains, thus disregarding important changes of monocrystalline magnetic anisotropy upon ferroelectric switching. The fact that increased magnetization is observed for polarization pointing away from Co (depletion of electrons) and the fast and energy-efficient response agree with the increase of spin offsetting at the Fermi level in the depletion state for the conventional magnetoelectric effect.^37,38^ This behavior, in addition to the absence of additional Co-oxidation upon switching (Figures S7–S9) and the characterized response time (<500 ns), is inconsistent with what would be expected from magnetoionic effects (see Table S1). To perform a more quantitative verification of the presence of the conventional charge-mediated magnetoelectric effect, we performed spin-polarized DFT calculations. For the sake of simplicity, we consider that the HfO_2_ layer adopts a polar phase with its electric polarization oriented downward (Figure 3a). For the relaxed equilibrium structure, the average magnetic moment of the Co atoms located at the HfO_2_/Co interface experiencing electrons depletion, induced by the direction of the ferroelectric polarization pointing downward and thus by the application of positive *V*_w_, was approximately 2.2 ± 0.1 μ_B_ per atom. In contrast, the Co atoms at the opposite polarization state, where electrons’ accumulation occurs, induced by the direction of the ferroelectric polarization pointing upward and thus by the application of negative *V*_w_, exhibit an average magnetic moment of 1.2 ± 0.1 μ_B_ per atom. These results demonstrate that the orientation and magnitude of the electric polarization in HfO_2_ can induce a substantial modulation of the magnetization depending on the polarization state, both enhancing and suppressing it, relative to bulk Co (1.5 μ_B_). As aforementioned, this behavior is consistent with previous first-principles studies on analogous HfO_2_/Ni system and can be further understood through an analysis of the partial electronic density of states (eDOS).^37,38^ Figure 3b,c presents the spin-resolved eDOS computed for Co atoms for two polarization directions, plotted as a function of energy relative to the Fermi level. The overall shapes of the spin-up and spin-down eDOS curves as well as their mutual energy splitting (≈2 eV) are similar at both interfaces. However, at the charge-depleted interface, both spin channels are shifted upward in energy by approximately 1 eV compared to that at the charge-accumulated interface. This energy shift leads to a larger imbalance between occupied spin-up and spin-down states at the charge-depleted interface, resulting in higher net magnetization for the depleted state. The predicted variation of the magnetization by our calculations is around 80%. In our system, this value must be scaled to the measured polarization, which is 15 μC/cm^2^ corresponding to the 25% of the polarization estimated for a phase pure HfO_2_ film (52–55 μC/cm^2^).^55,56^ The reason for this decreased measured polarization is 2-fold: (i) orthorhombic ferroelectric grains coexist with nonferroelectric monoclinic grains as revealed by the Scanning Transmission Electron Microscopy (STEM) image shown in Figure 3d (see Figure S10 for phase identification) and (ii) polarization is tilted by ≈55° with respect to the normal axis of the film, as represented by the orange arrow shown in Figure 3d. These two factors would indicate that a 20% increase of magnetization instead of 80% must be expected. Additionally, the Co thickness variation across the film as evidenced in the near 1 μm lateral size High-Angle Annular Dark Field (HAADF)-STEM image of Figure 3e, the presence of oxygen at the bulk of the Co layer (Figure S11), and the fact that DFT magnetization change calculation is done for the first Co layer and MOKE probes the whole 1.5 nm Co layer further account for the decreased measured 5% variation. Note that the fact that XAS-PEEM is a surface-sensitive technique indicates that the Co-oxidation is not very relevant and probably limited to the interface with La:HZO. Therefore, DFT calculations along with structural characterization indicate that the 5% variation of magnetization is in good agreement with the presence of conventional magnetoelectric effect. Larger relative responses could be achieved by reducing the Co thickness; however, the possible decrease in the interface quality may be detrimental. The performed characterization also aligns with the absence of direct evidence of electronically driven changes in magnetization in polycrystalline HfO_2_ systems,^39–41^ in which the presence of blurry interfaces and larger metallic magnetic oxidation can be expected, probably hindering the present conventional magnetoelectric effects.

**Figure 3:**
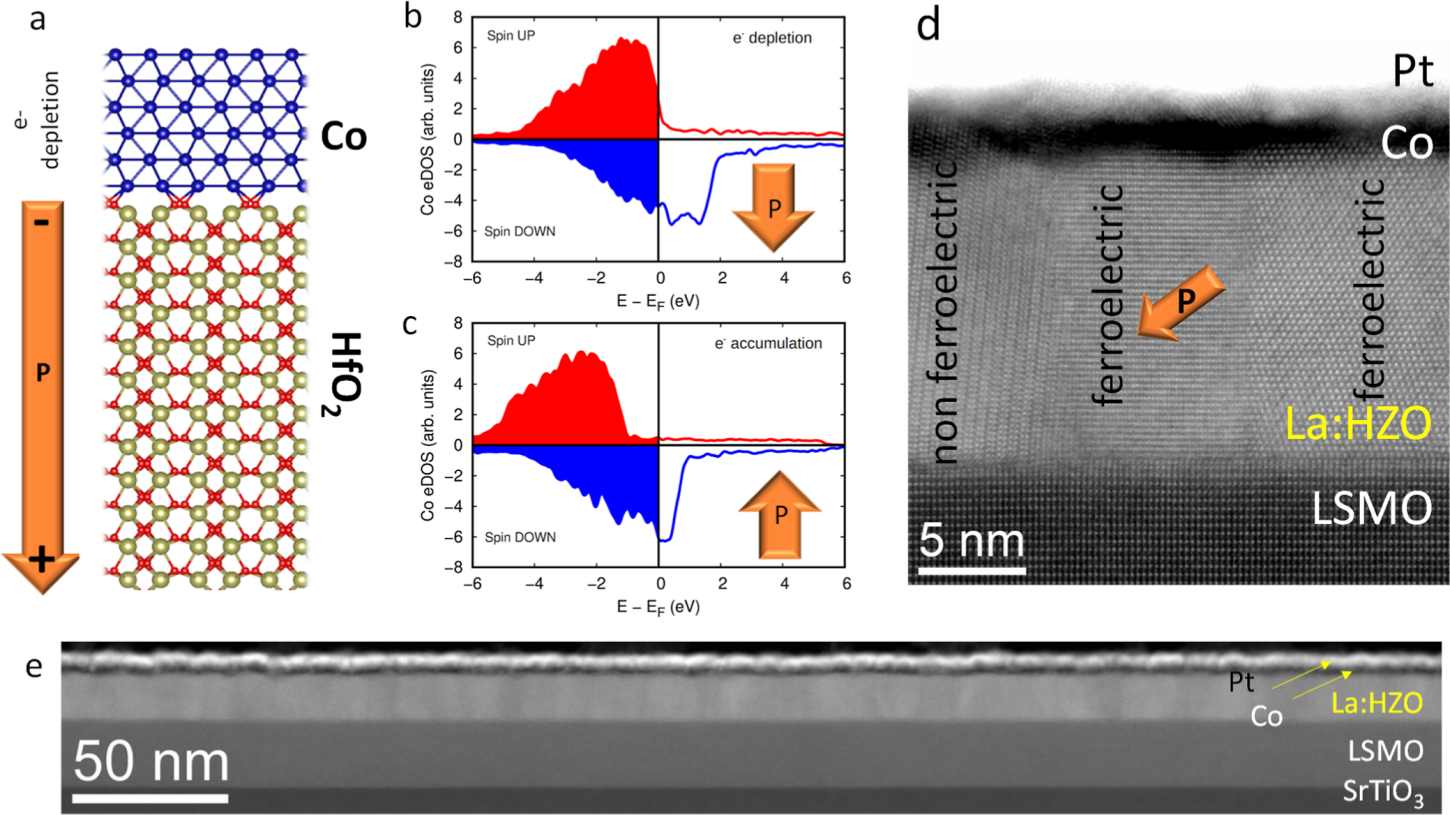
(a) Sketch of the simulated first-principles spin-polarized DFT calculations Co/HfO_2_ system where HfO_2_ adopts a polar phase with the polarization pointing downward. Blue, gold, and red spheres represent Co, Hf, and O atoms, respectively. (b) Partial density of electronic states calculated for the Co atoms located in the Co/HfO_2_ interface where the electronic charge is depleted. (c) Idem for accumulation state. (d) Representative high-magnification image STEM image. (e) Low-magnification HAADF-STEM cross-sectional image.

### Multilevel Modulation of Magnetization

Ferroelectrics allow multilevel polarization states^57,58^ arising from the different balance between up and down domains after electrical stimuli of varying amplitude and duration. However, direct evidence of magnetic multistate behavior in systems exhibiting conventional magnetoelectric coupling has not been reported, despite their potential interest for beyond-von Neumann neuromorphic computing, where the simultaneous analog modulation of ferroelectric polarization and magnetization could enable new analog memory devices with combined functionalities based on CMOS-compatible materials. In Figure 4, we show the multilevel modulation of ferroelectric polarization and the Co magnetization by partial switching in two different ways, either by using a reduced voltage or by using shorter duration pulses than those required for saturation. In Figure 4a, a simplified sketch of the voltage train pulse used to modulate the ferroelectric polarization and the magnetic states using different *V*_w_ values is plotted. Precisely, devices are saturated in the upward state (negative voltage pulse not shown), afterward a voltage pulse indicated in the legend *V*_w_ is applied and finally a positive triangular signal of 1 kHz and 8 V is used to read the polarization state and plotted in Figure 4b. The same has been realized for the opposite polarization state, and it is also shown in Figure 4b. Figure 4b shows that switched polarization varies with *V*_w_ in a gradual manner using τ_w_ = 10 μs. Figure 4c shows the dependence of the switched polarization versus *V*_w_ for both polarities greatly resembling the variation of the Δ*M* signal shown in Figure 4d. In fact, there is a good correlation between Δ*M* and polarization, as shown in Figure S12, again disregarding important redox contributions. In Figure 4e, a sketch of the voltage train pulse used to modulate the ferroelectric polarization and the magnetic states using increasing τ_w_ with fix |*V*_w_| = 8 V is plotted. Figure 4f–h shows the gradual variation of polarization and Δ*M* with τ_w_. It can be observed that Δ*M* (Figure 4h) can be modulated analogously to the ferroelectric polarization (Figure 4g). This multilevel response arises from the varying balance between up and down ferroelectric domains, which, in turn, leads to a corresponding variation between regions of higher and lower magnetization. Note that the Δ*M* value systematically saturates at 5% (Figure 4d,h) indicating the good reproducibility of the found effect. The fact that Δ*M* smoothly follows the polarization response and saturates well below the 100% level expected for full oxidation of the magnetic layer in comparable systems (Supporting Information Table S1)^5–8^ provides further evidence that the observed magnetization changes are driven by ferroelectric polarization rather than magneto-ionic effects. Finally, we emphasize that Δ*M* can be measured after a 1–5 days interval after the electric-field pulse. This is possible owing to the very high retention of the samples as shown in Figure S13. Indeed, extrapolated polarization retention in epitaxial ferroelectric hafnia crystallized at high temperature is systematically observed to exceed 10 years at 85 °C.^59^ These previous results are also reproduced in the devices characterized here (Figure S13). In addition, magnetization is primarily employed as a storage parameter due to its long-term stability.^60^ Both facts support the notion that the retention of the magnetoelectric response is expected to be robust in a prospective magnetoelectric computing device.

**Figure 4:**
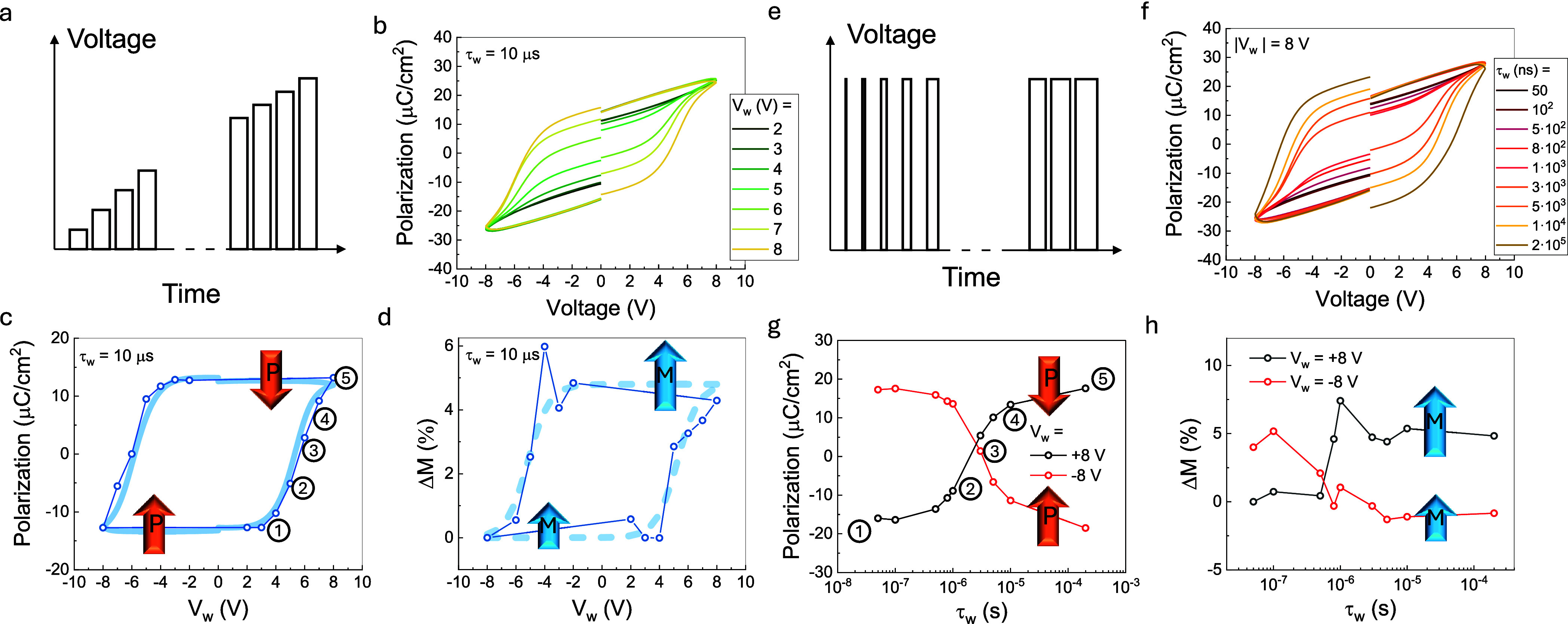
(a) Schematics of the voltage pulses of different *V*_w_. (b) Reading polarization curves of indicated *V*_w_ with both polarities. (c) Polarization versus *V*_w_. (d) Δ*M* versus *V*_w_. (e) Schematics of the voltage pulses of different τ_w_. (f) Reading polarization curves of indicated τ_w_ with both polarities. (g) Polarization versus τ_w_. (h) Δ*M* versus τ_w_.

## Conclusions

In this work, we demonstrate electric-field manipulation of magnetization in a Co/La:HZO heterostructure at room temperature with a response time faster than 500 ns and low power consumption. The presented results agree with conventional magnetoelectric coupling, where polarization reversal causes a modification of the spin-dependent asymmetry in the density of states of the Co layer. No evidence of magneto-ionic effects is obtained from XAS characterization (oxidation/reduction of Co layer upon electric-field application), which enables the observed fast response time and low energy consumption. We also demonstrate the possibility of gradually tuning the magnetization, paving the way to develop neuromorphic post-von Neumann devices based on a system that exhibits high ferroelectric and ferromagnetic Curie temperatures.

## Methods

### Device Fabrication

La:HZO films and bottom LSMO electrodes were grown in a single process by using pulsed laser deposition with a KrF excimer laser. Sintered La(1%):Hf_0.5_Zr_0.5_O_2–*x*_ and La_0.67_Sr_0.33_MnO_3_ ceramics were used as targets. LSMO electrodes were deposited at a substrate temperature of 700 °C, an oxygen pressure of 0.1 mbar, and a laser frequency of 5 Hz. For the growth of La:HZO films, the corresponding parameters were 2 Hz, 800 °C, and 0.1 mbar. Thickness of La:HZO is 15 nm, as determined from Laue fringes simulation, as shown in Figure S1 and in agreement with STEM characterization (Figure 3). Square 60 × 60 μm^2^ patterns (Figure 1a, inset) comprising Pt (3 nm) on top of Co (1.5 nm) were grown ex situ by DC magnetron sputtering at room temperature onto the La:HZO films through stencil masks.

### Structural, Electric, and Ferroelectric Characterization

Crystal structure analysis was performed using X-ray diffraction with Cu K_α_ radiation by employing a Bruker Discovery diffractometer equipped with a point detector. Ferroelectric polarization loops, τ_w_-dependent, *V*_w_-dependent, leakage, endurance, and retention, were measured at room temperature using an AixACCT TFAnalyser3000 platform, with the LSMO bottom electrode connected to the ground and bias applied to the top Pt/Co structure. Ferroelectric polarization loops were obtained in dynamic leakage current compensation mode with a frequency of 1 kHz.^61^ Endurance properties were demonstrated by ferroelectric polarization measurements, as shown in Figure S14. In all the carried-out experiments, voltage has been applied to the top Pt/Co layer, while the LSMO layer is grounded. Leakage current was measured using the same platform and an integration time of 1 s. PFM measurements were performed with an MFP-3D microscope (Oxford Instruments Co.) using BudgetSensors silicon (n-type) cantilevers with Pt coating (Multi75E-G). To enhance sensitivity, the dual AC resonance tracking method was employed.^62^ To remove charging effects contribution, additional bias voltage was also employed during PFM characterization.^63^

### Magnetic and Magnetoelectric Characterization

The electrical pulses were applied prior to magnetic characterization (1–5 days in advance), indicating good retention of the magnetoelectric effect. The evolution of the local magnetic properties was investigated using a Durham Magneto Optics Ltd. polar MOKE apparatus with a laser focused to a spot size of ≈3 μm. The MOKE experiments were performed by applying an in-plane magnetic field (*H*_app_ < 500 Oe). The amplitude of the Kerr rotation θ_K_ (i.e., MOKE signal) is roughly proportional to the longitudinal in-plane magnetic moment of the film and ultimately to its magnetization (*M*): θ_K_(*H*_app_) ∝ *m*(*H*_app_) ∝ *M*(*H*_app_).^64^

XMCD-PEEM experiments were performed at the CIRCE beamline of the ALBA Synchrotron^65^ using circularly polarized X-ray with an energy resolution of *E*/Δ*E* ≈ 5000. Before imaging, the Pt capping layer was removed to obtain a better magnetic contrast. All images were recorded at the Co L_3_ edge at ≈777 eV. The field of view was 40 μm. Electric-field pulses were applied ex situ by the AixACCT TFAnalyser3000 platform in different devices. Two different samples grown under nominally the same conditions were used to perform magnetoelectric characterization by MOKE and XMCD-PEEM. Saturation polarization state was ensured by the application of τ_w_ = 2 ms and *V*_w_ = −8 or +8 V pulses. XMCD contrast is obtained by averaging the peak positions shown in Figure 2e.

### First-Principles Calculations

First-principles calculations based on spin-polarized DFT^66^ were carried out with the PBEsol exchange–correlation energy functional^67^ as it is implemented in the VASP software.^68^ The “projector-augmented wave” method^69^ was employed to represent the ionic cores by considering the following electronic states as valence: Hf 5d6s5p; O 2s2p; Co 3d4s. An energy cutoff of 750 eV and a dense Monkhorst–Pack *k*-point density (equivalent to that of a 12 × 12 × 12 grid for the 12-atom bulk HfO_2_ unit cell) was used for integration within the Brillouin zone, leading to total energies converged to within 1 meV per formula unit. Atomic relaxations were concluded when the forces in all of the atoms were below 0.005 eV/Å. The simulated Co/HfO_2_/Co system contained a total of 120 atoms (48 Co, 48 O, and 24 Hf ions) with a length of approximately 50 Å along the direction perpendicular to the HfO_2_/Co interfaces. All of the atomic positions were fully relaxed in the simulations. Additionally, the shape of the simulation cell was optimized while constraining the volume of the full slab, which includes a vacuum region. This approach minimizes the elastic penalty associated with the formation of the HfO_2_/Co interface. The resulting relaxed supercell dimensions are 5.20 × 4.94 × 76.25 Å. For the sake of simplicity, the simulated system has a downward defined direction, and the top HfO_2_/Co interface is taken as the one corresponding to upward polarization and the bottom interface as the one for downward interface as shown in Figure S15. A vacuum region of approximately 25 Å thickness was added to the simulation supercell along the same direction.

### Scanning Transmission Electron Microscopy

Atomic-scale structural analysis of selected films was performed by STEM in HAADF imaging mode. A Thermo Fisher Titan 60-300 microscope equipped with a high brightness Schottky field emission gun and a CETCOR probe-corrector (CEOS GmbH) was operated at 300 kV to provide a probe size below 0.1 nm. X-ray energy-dispersive spectroscopy (EDS) was performed in an Ultim Max TLE10 spectrometer from Oxford Instruments. Cross-sectional lamellae of the specimens, cut along (110) and (100) planes of the STO substrate, were prepared by Focused Ion Beam milling in a Thermo Fisher Helios 650 Nanolab. STEM image simulations were carried out with the Dr. Probe software package.^70^

## Supplementary Material





## Data Availability

The data that support the findings of this study are available from the corresponding author upon request.

## Notes

Additional structural, compositional, and ferroelectric characterization (PDF)
Isotropic domains that agree with the 4 crystalline variants and 3 different possible directions for the ferroelectric polarization vector of La:HZO, resulting in an expected lack of anisotropy along in-plane directions (MP4)
