# Supplementary material for: Electronically Driven Magnetoelectric Coupling in Co/La:Hf0.5Zr0.5O2 Heterostructures for Energy-Efficient Neuromorphic Computing
Source: ACS Appl Mater Interfaces. 2026 Feb 25;18(9):13970–9. doi: 10.1021/acsami.5c22784 (PMC12983206; doi:10.1021/acsami.5c22784)
Supplement: Supplementary file 1 [file am5c22784_si_001.pdf]

Supporting Information

Electronically Driven Magnetoelectric Coupling in Co/La:$Hf_{0.5}Zr_{0.5}O_2$ Heterostructures for Energy Efficient Neuromorphic Computing

Alberto Quintana,[1,*] Cesar Magen,[2,3] Mehrdad Ghiasabadi Farahani,[1] Wenjing Dong,[1] Jingye Zou,[1] Nico Dix,[1] Zheng Ma,[4] Enric Menéndez,[4] Michael Foerster,[5] Miguel Angel Niño,[5] Claudio Cazorla,[6,7,8] Jordi Sort,[4,8,9] Florencio Sánchez,[1,*] Ignasi Fina[1,*]

[1]Institut de Ciència de Materials de Barcelona (ICMAB-CSIC), Campus UAB, Bellaterra 08193, Barcelona, Spain
[2]Instituto de Nanociencia y Materiales de Aragón (INMA), CSIC-Universidad de Zaragoza, 50009 Zaragoza, Spain
[3]Departamento de Física de la Materia Condensada, Universidad de Zaragoza, 50018 Zaragoza, Spain
[4]Departament de Física, Universitat Autònoma de Barcelona, 08193 Cerdanyola del Vallès, Spain
[5]Consorci per a la Construcció, Equipament i Explotació del Laboratori de Llum Sincrotró, Divisió Experiments, Physics Section.
[6]Group of Characterization of Materials, Departament de Física, Universitat Politècnica de Catalunya, Campus Diagonal Besòs, Av. Eduard Maristany 10–14, 08019 Barcelona, Spain
[7]Research Center in Multiscale Science and Engineering, Universitat Politècnica de Catalunya, Campus Diagonal-Besòs, Av. Eduard Maristany 10–14, 08019 Barcelona, Spain
[8]Institució Catalana de Recerca i Estudis Avançats (ICREA), Pg. Lluís Companys 23, E-08010 Barcelona, Spain.
[9]Catalan Institute of Nanoscience and Nanotechnology (ICN2), CSIC and BIST, E-08193 Barcelona, Spain.
**Corresponding authors' e-mail: alberto.quintana@uab.cat, fsanchez@icmab.es, ifina@icmab.es.*

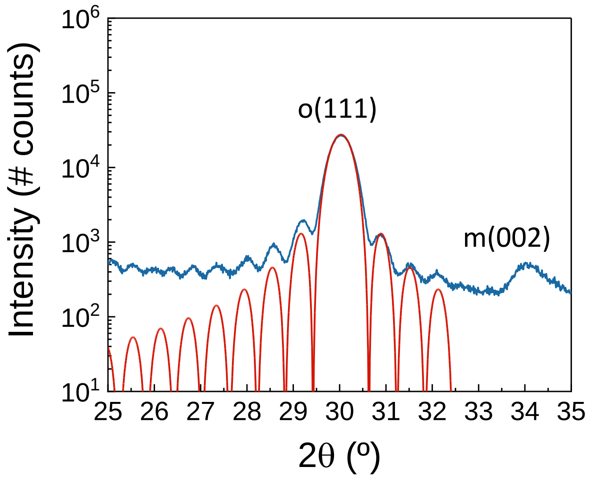

Figure S1. XRD θ–2 θ scan of the deposited films. Lines correspond to simulation of Laue fringes. The peak visible near 30° corresponds to the position of the (111) reflection of o-phase. The peak visible near 34° corresponds to the position of the (002) reflection of the monoclinic phase. The less significative presence of non-ferroelectric monoclinic phase is confirmed by the TEM characterization.

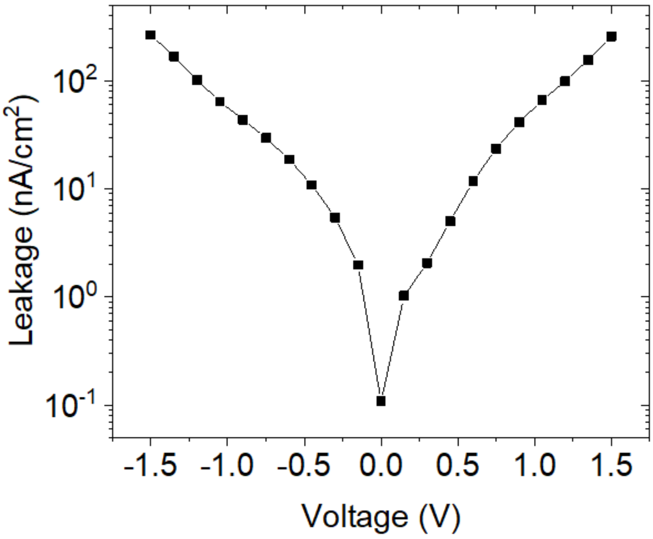

Figure S2. Leakage current obtained using 1s integration time. Low leakage current can be inferred from this experiment.

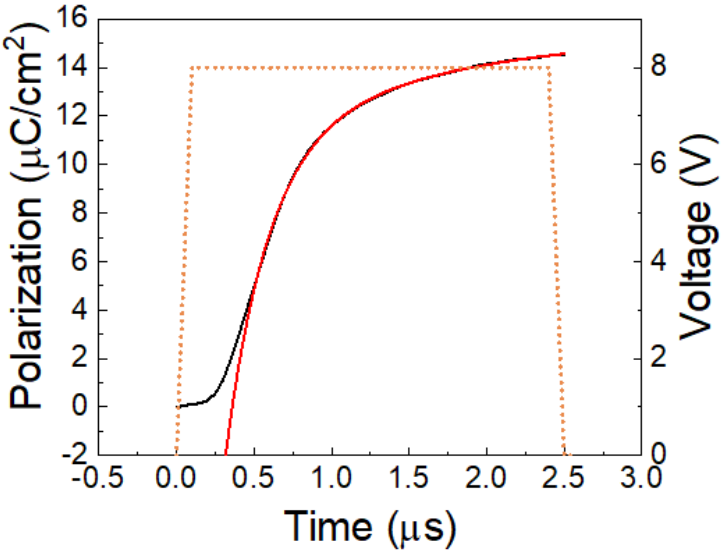

Figure S3. Polarization integrated during the application of the pulse shown in Figure 1a of the main text.

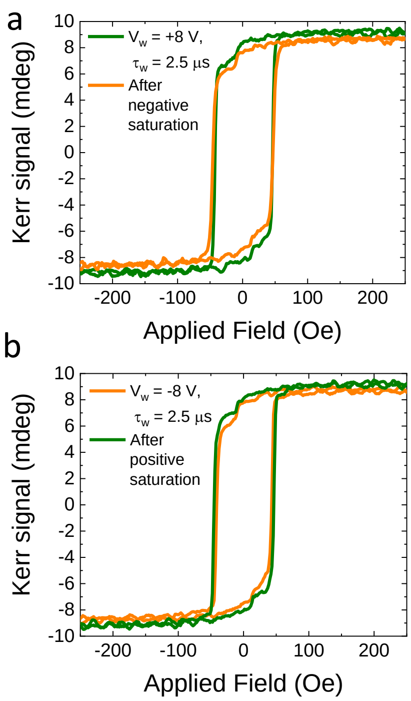

Figure S4. a,b. Kerr magnetic loops after saturation and subsequent V_w = +8, -8 V voltage pulses of τ_w = 2.5 μs, respectively. The data were collected using the same procedure as for Figure 1c,d. The close resemblance of the results indicates their good reproducibility.

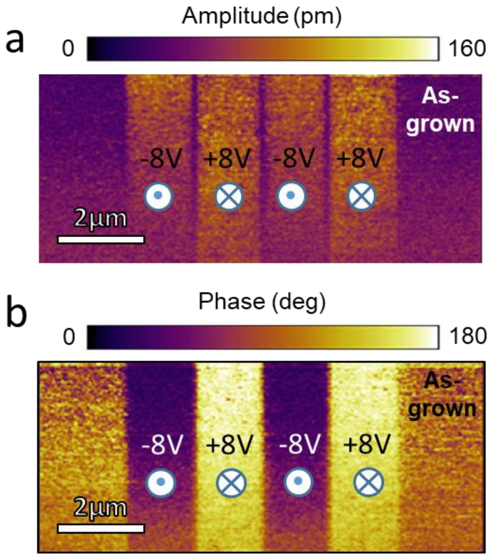

Figure S5. PFM a, amplitude and b, phase image of the La:HZO surface after poling with indicated voltage and in the as-grown state. It can be observed that the contrast of the as grown state is similar that after application of +8 V, indicating that the as-grown state is mainly downwards.

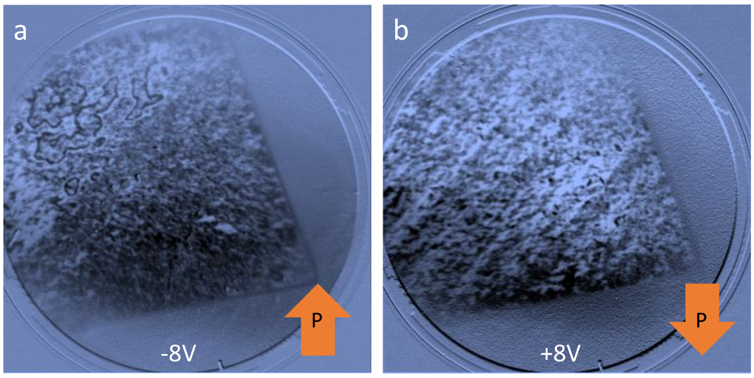

Figure S6. XMCD-PEEM images in after application of a. V_w = -8 V and b. V_w = +8 V with τ_w = 2.5 μs in devices different to those shown in Figure 2 of the main text.

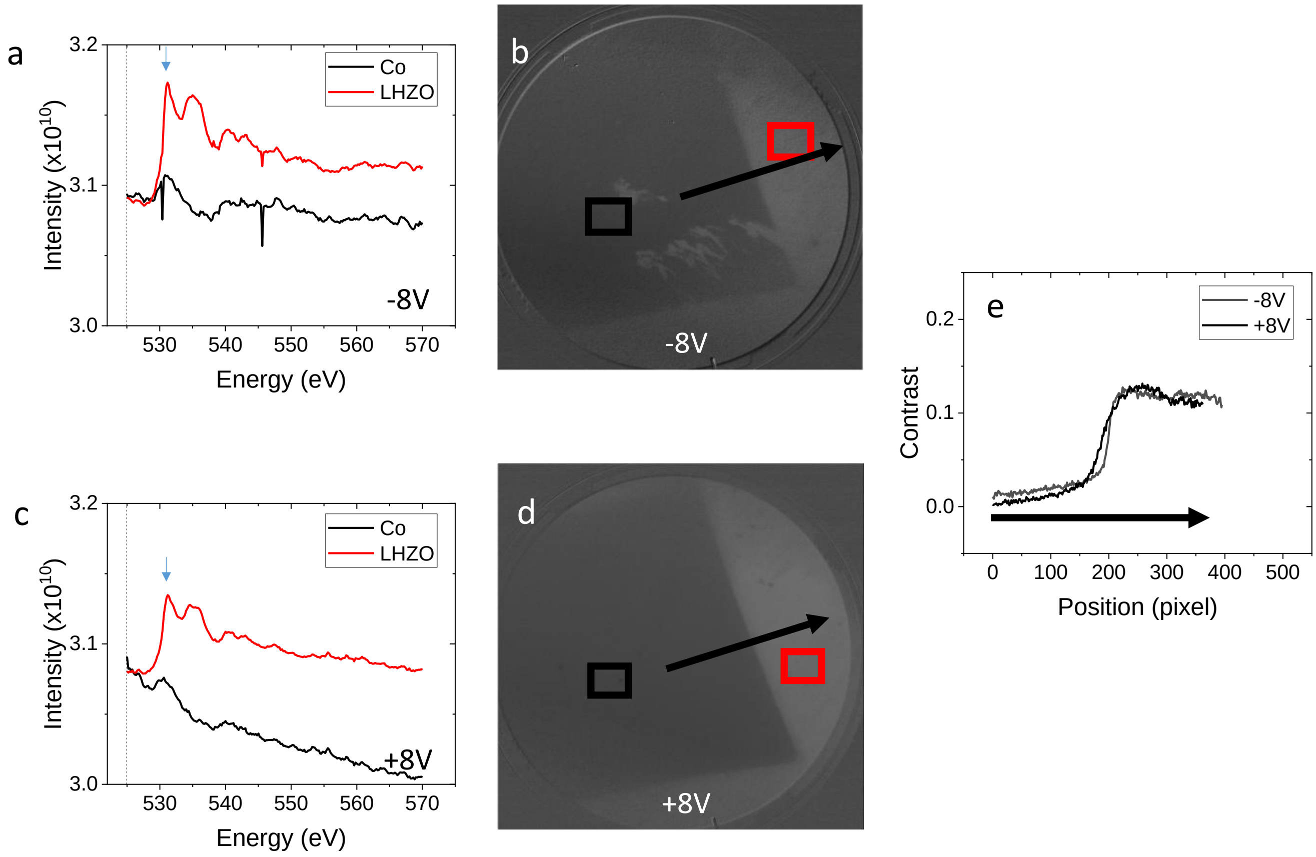

Figure S7. a,c. XAS spectra of the regions shown in the panels b,d, respectively, and b,d. XAS images at 531 eV . e. Contrast profile along the arrows shown in panels b,d after indicated polarity. The similar contrast of images b,d, as clearly shown in panel e, indicates absence of important change of oxygen state/amount.

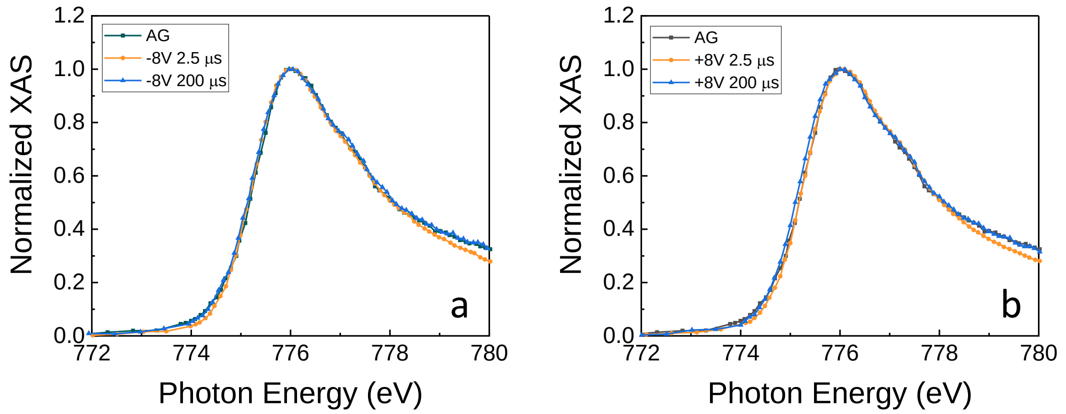

Figure S8. a,b. XAS spectra near Co L_3 edge for indicated voltage polarity and as grown (AG). The absence of any important variation at the right of the peak (near 777 eV) for different polarity indicate that Co has not been significantly oxidized/reduced upon the application of the different voltages.

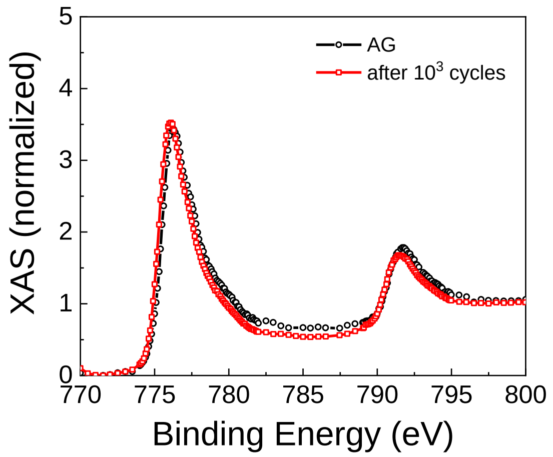

Figure S9. XAS spectra near Co L_3 edge for as grown (AG) and after electrical cycling of 10^3 cycles at 8 V. The absence of any important variation at the right of the peak (near 777 eV) for different polarity indicate that Co has not been significantly oxidized/reduced upon the application of the electrical cycling.

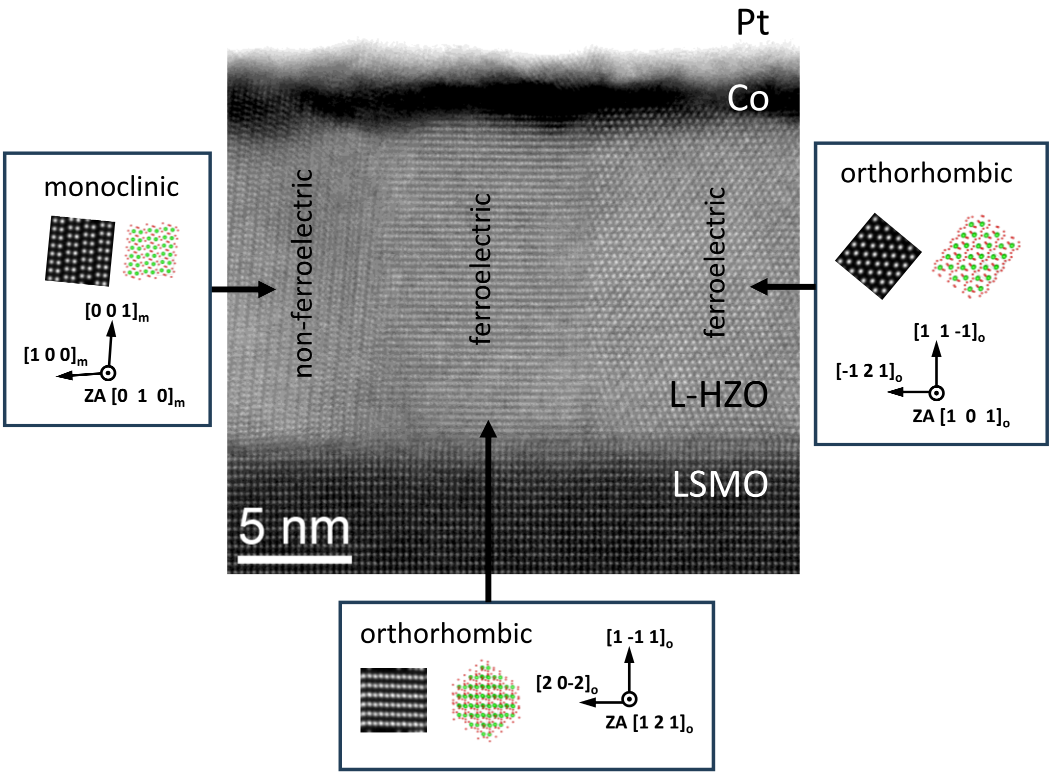

Figure S10. Representative high magnification image STEM image of Figure 3e together with simulations of the expected contrast for the indicated phases and orientations, which served for phase identification in each grain.

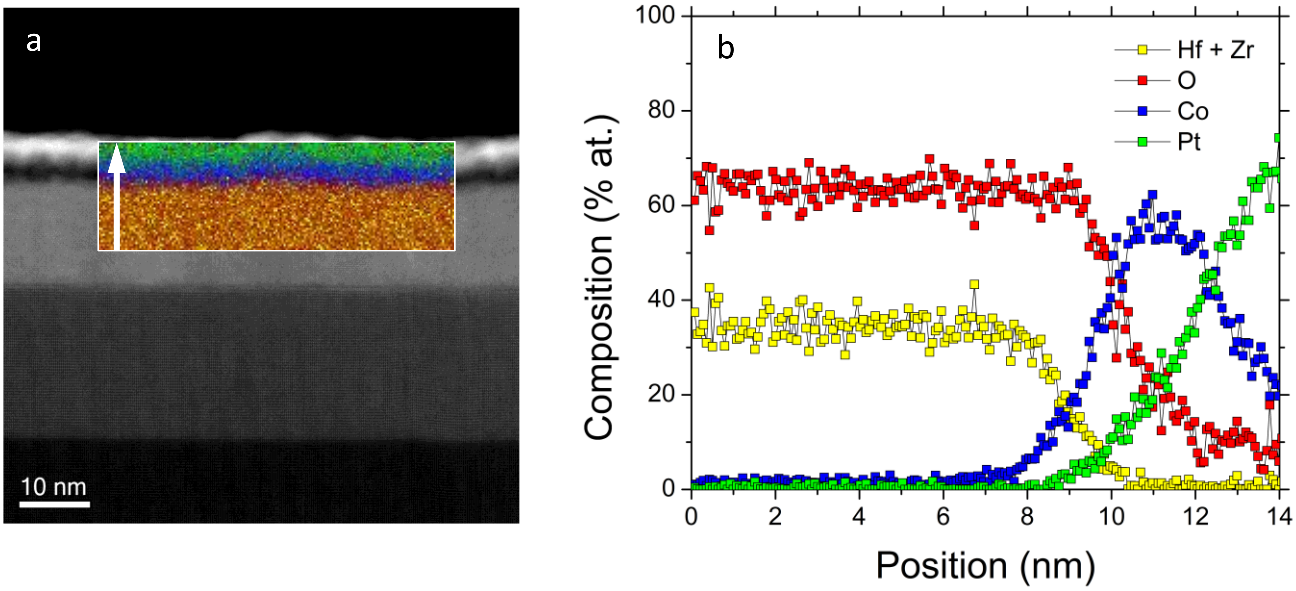

Figure S11. STEM-EDS analysis of the HZO-Co interface. a. HAADF-STEM image with an inset of the EDS chemical map in color (Hf+Zr in yellow, oxygen in red, cobalt in blue, platinum in green). b. Cross sectional chemical line profile obtained by integrating approximately 10 nm of the region mapped in a, with the same color code.

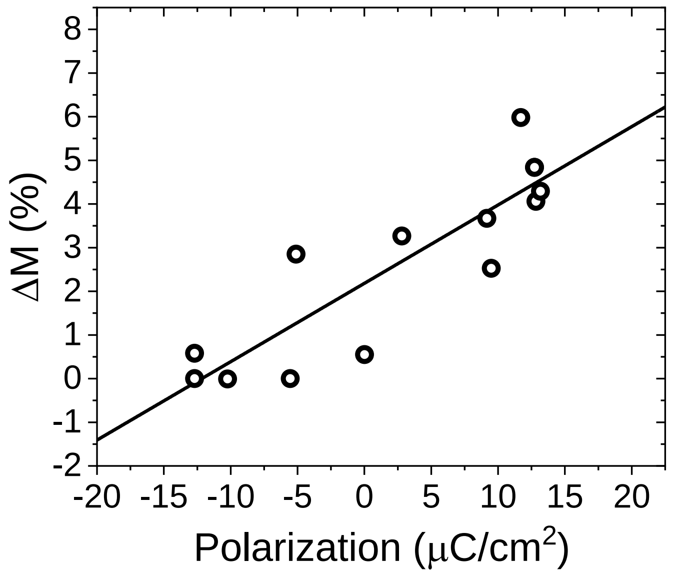

Figure S12. Dependence of ΔM on Polarization extracted from data in Figure 4c,d. The good correlation between magnetization and polarization indicates the coupling between these two magnitudes.

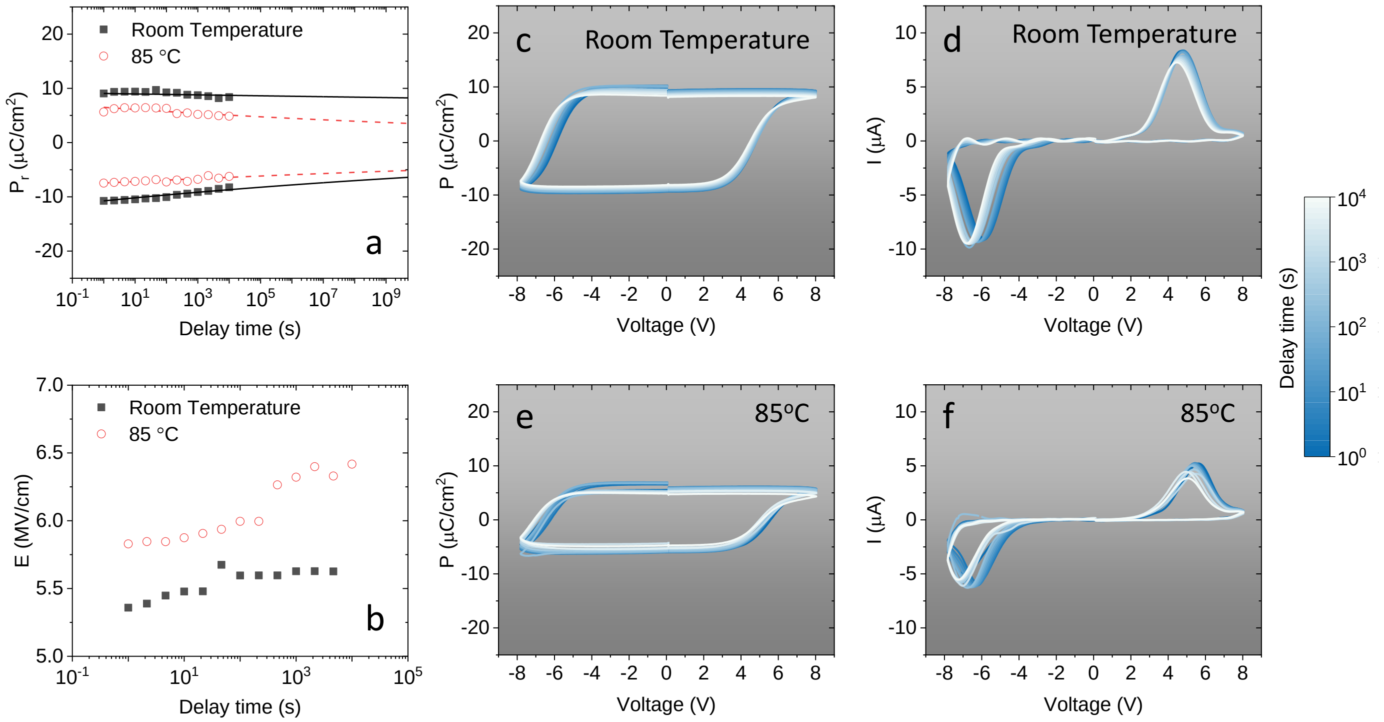

Figure S13. a,b. Polarization and coercive electric field retention data, respectively, at room temperature and 85°C. c,d. Polarization (P) and current (I) during the PUND reading in the retention experiments at room temperature. e,f. Polarization (P) and current (I) during the PUND reading in the retention experiments at 85°C. The similarity of the reading P and I indicates the good retention of the films. Data in panels c-f is obtained after electric poling and delay time indicated in the legend.

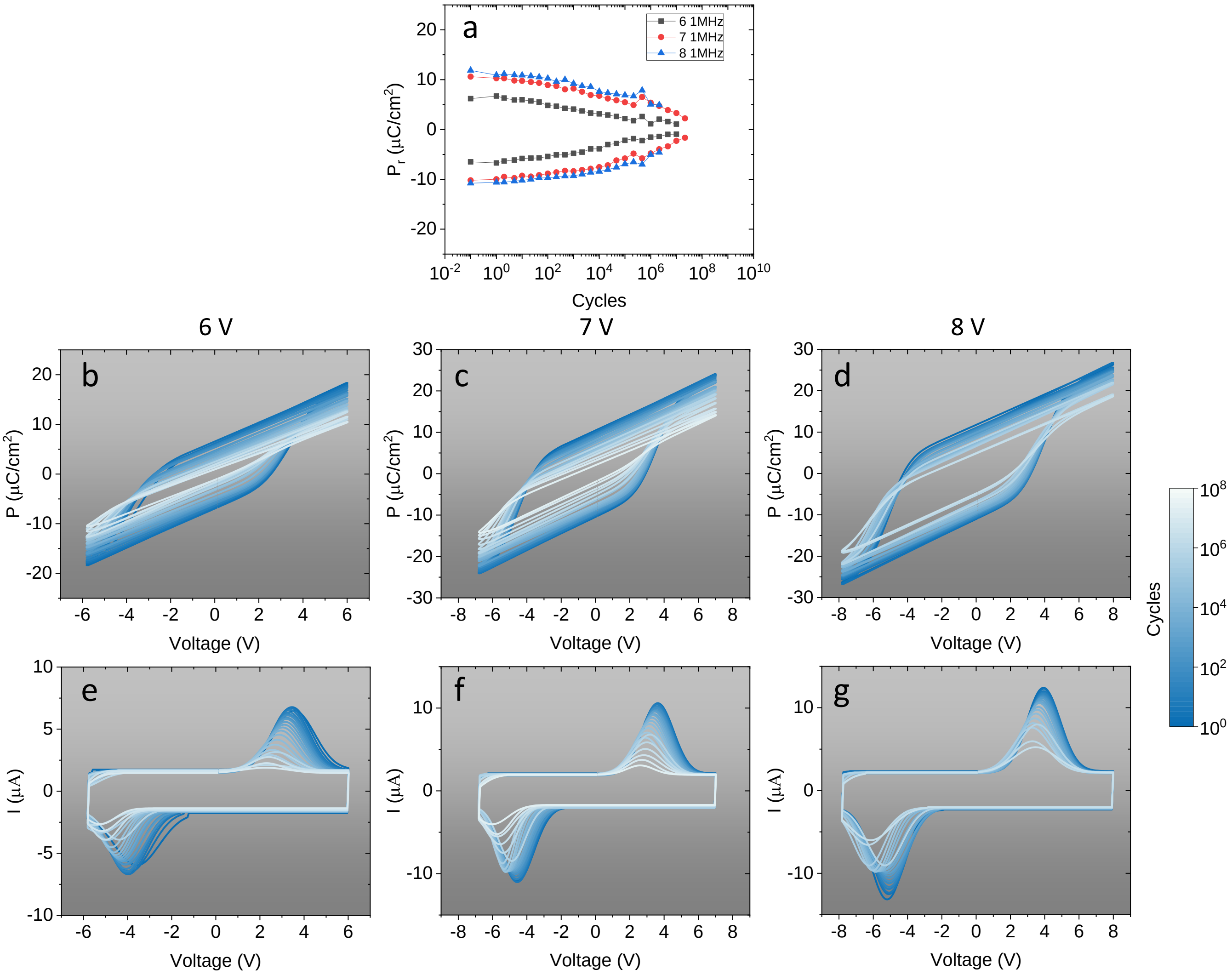

Figure S14. a. Endurance data for indicated cycling voltage at cycling frequency of 1 MHz. b,c,d. Polarization (P) and e,f,g current (I) loops after leakage compensation for 6, 7 and 8 V cycling voltage amplitude, respectively. Note that polarization values in the pristine loops are slightly lower to those shown in Figure 3,4 due to leakage compensation. The data indicates sizable polarization even after the application of 10^7 cycles.

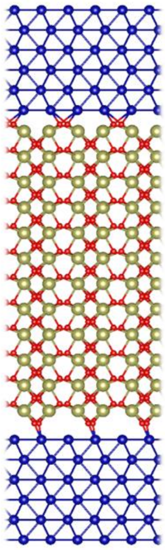

Figure S15. Simulated structure.

Table S1. Summary of representative results of the literature. TMR: Tunnel magnetoresistance. Hc: Coercive Magnetic Field. EB: Exchange Bias. VSM: Vibrating-sample magnetometer. BTO: $BaTiO_3$. HZO: $Hf_{0.5}Z_{0.5}rO_2$. BFO: $BiFeO_3$. Empty cells indicate that information is not provided. Reference number corresponds to the reference list of the main text. It can be inferred that representative examples of systems where changes in magnetization are mediated by magnetoionic effects, the sign of the change of magnetization is always negative. In the case of systems based on ferroelectric materials, the sign of the change of magnetization, if defined, show dispersive results, which indicates the coexistence of several mechanism. The positive sign, regarding the change of magnetization with voltage, and the fast response time found in our systems, indicate the absence of ionic migration effects in the results of the present work. ND: Not defined. NR: Not reported.

Type	Structure	ΔM/M (%)	Detection technique / Magnitude	Sign	Response time	Multi-level	Mechanism	Ref.
Ionic conductors	Pt/Co/GdO_x	100%	MOKE	negative*	10 ms	Y	magnetoionic	1
	Pt/Co/MoOx/ZrO_2	100%	MOKE	negative	10 ms	N	magnetoionic	2
	Co/Pd/YSZ	100%	MOKE	negative	10 ms	Y	magnetoionic	3
	Pt/Co/GdO_x	100%	Hall/MOKE	negative	100 ms	Y	magnetoionic	4
Ferroelectric perovskite based	Fe/BTO	25%	Hc	ND	NR	Y	strain	5
	BFO/LSMO	ND	EB sign	not defined	NR	N	AFM-FM transition	6
	Ta/Co/BFO	60%	Hc	negative	100 ns	Y	redox	7
	Pt/Co/P(VDF-TrFE)	30%	Hc	negative	NR	Y	conventional/charge doping*	8-9
	CoFe/BFO	ND	MFM	ND	NR	N	AFM/FM magnetic coupling	10
	FeRh/PMN-PT	34%	VSM/XMCD	symmetric	1	Y	strain	11-15
Ferroelectric HfO_2 - based	Ni/HfO_2	25%	theory	positive	NR	N	conventional/ charge doping	16-17
	Ni/HfO_2	25%	theory	negative	NR	N	redox	16-17
	Ni/HZO	0.2	XAS	ND	NR	N	redox	18
	Co/Al:HfO_2	negligible	Hall	positive	NR	Y	conventional/charge doping	19
	Co/HZO	negligible	TMR	ND	NR	N	conventional/charge doping	20
	Co/HZO	TMR reversed sign	TMR	ND	NR	N	redox	20
	**Co/La:HZO**	**5%**	**MOKE/ XMCD**	**positive**	**< 500 ns**	**Y**	**conventional/charge doping**	**Present work**

Supporting Information reference list

(1) Bauer, U.; Yao, L.; Tan, A. J.; Agrawal, P.; Emori, S.; Tuller, H. L.; van Dijken, S.; Beach, G. S. D. Magneto-Ionic Control of Interfacial Magnetism. *Nat. Mater.* **2015,** *14* (2), 174-181.
(2) Fassatoui, A.; Ranno, L.; PeñaGarcia, J.; Balan, C.; Vogel, J.; Béa, H.; Pizzini, S. Kinetics of Ion Migration in the Electric Field-Driven Manipulation of Magnetic Anisotropy of Pt/Co/Oxide Multilayers. *Small* **2021,** *17* (38), 2102427.
(3) Lee, K.-Y.; Jo, S.; Tan, A. J.; Huang, M.; Choi, D.; Park, J. H.; Ji, H.-I.; Son, J.-W.; Chang, J.; Beach, G. S. Fast Magneto-Ionic Switching of Interface Anisotropy Using Yttria-Stabilized Zirconia Gate Oxide. *Nano Lett.* **2020,** *20* (5), 3435-3441.
(4) Tan, A. J.; Huang, M.; Avci, C. O.; Büttner, F.; Mann, M.; Hu, W.; Mazzoli, C.; Wilkins, S.; Tuller, H. L.; Beach, G. S. D. Magneto-Ionic Control of Magnetism Using a Solid-State Proton Pump. *Nat. Mater.* **2019,** *18* (1), 35-41.
(5) Sahoo, S.; Polisetty, S.; Duan, C.-G.; Jaswal, S. S.; Tsymbal, E. Y.; Binek, C. Ferroelectric Control of Magnetism in $BaTiO_3$/Fe Heterostructures Via Interface Strain Coupling. *Phys. Rev. B* **2007,** *76* (9), 092108.
(6) Wu, S. M.; Cybart, S. A.; Yi, D.; Parker, J. M.; Ramesh, R.; Dynes, R. C. Full Electric Control of Exchange Bias. *Phys. Rev. Lett.* **2013,** *110* (6), 067202.
(7) Chen, Z.; Sun, H.; Zhou, X.; Duan, H.; Yan, W.; Yin, Y.; Li, X. Continuous and Fast Magneto-Ionic Control of Magnetism in Ta/Co/$BiFeO_3$/$SrRuO_3$ Multiferroic Heterostructure. *J. Materiomics* **2022,** *8* (6), 1141-1148.
(8) Mardana, A.; Ducharme, S.; Adenwalla, S. Ferroelectric Control of Magnetic Anisotropy. *Nano Lett.* **2011,** *11* (9), 3862-3867.
(9) Huang, Z.; Stolichnov, I.; Bernand-Mantel, A.; Borrel, J.; Auffret, S.; Gaudin, G.; Boulle, O.; Pizzini, S.; Ranno, L.; Herrera Diez, L.; Setter, N. Ferroelectric Control of Magnetic Domains in Ultra-Thin Cobalt Layers. *Appl. Phys. Lett.* **2013,** *103* (22), 222902
(10) Vaz, D. C.; Lin, C.-C.; Plombon, J. J.; Choi, W. Y.; Groen, I.; Arango, I. C.; Chuvilin, A.; Hueso, L. E.; Nikonov, D. E.; Li, H.; Debashis, P.; Clendenning, S. B.; Gosavi, T. A.; Huang, Y.-L.; Prasad, B.; Ramesh, R.; Vecchiola, A.; Bibes, M.; Bouzehouane, K.; Fusil, S.; Garcia, V.; Young, I. A.; Casanova, F. Voltage-Based Magnetization Switching and Reading in Magnetoelectric Spin-Orbit Nanodevices. *Nat. Commun.* **2024,** *15* (1), 1902.
(11) Cherifi, R. O.; Ivanovskaya, V.; Phillips, L. C.; Zobelli, A.; Infante, I. C.; Jacquet, E.; Garcia, V.; Fusil, S.; Briddon, P. R.; Guiblin, N.; Mougin, A.; Ünal, A. A.; Kronast, F.; Valencia, S.; Dkhil, B.; Barthélémy, A.; Bibes, M. Electric-Field Control of Magnetic Order above Room Temperature. *Nat. Mater.* **2014,** *13* (4), 345-351.
(12) Clarkson, J. D.; Fina, I.; Liu, Z. Q.; Lee, Y.; Kim, J.; Frontera, C.; Cordero, K.; Wisotzki, S.; Sanchez, F.; Sort, J.; Hsu, S. L.; Ko, C.; Aballe, L.; Foerster, M.; Wu, J.; Christen, H. M.; Heron, J. T.; Schlom, D. G.; Salahuddin, S.; Kioussis, N.; Fontcuberta, J.; Marti, X.; Ramesh, R. Hidden Magnetic States Emergent under Electric Field, in a Room Temperature Composite Magnetoelectric Multiferroic. *Sci. Rep.* **2017,** *7* (1), 15460.
(13) Fina, I.; Quintana, A.; Padilla-Pantoja, J.; Marti, X.; Macia, F.; Sanchez, F.; Foerster, M.; Aballe, L.; Fontcuberta, J.; Sort, J. Electric-Field-Adjustable Time-Dependent Magnetoelectric Response in Martensitic FeRh Alloy. *ACS Appl. Mater. Interf.* **2017,** *9* (18), 15577-15582.
(14) Fina, I.; Quintana, A.; Martí, X.; Sánchez, F.; Foerster, M.; Aballe, L.; Sort, J.; Fontcuberta, J. Reversible and Magnetically Unassisted Voltage-Driven Switching of Magnetization in FeRh/PMN-PT. *Appl. Phys. Lett.* **2018,** *113* (15), 152901.
(15) Fina, I.; Fontcuberta, J. Strain and Voltage Control of Magnetic and Electric Properties of FeRh Films. *J. Phys. D: Appl. Phys.* **2020,** *53* (2), 023002.
(16) Yang, Q.; Tao, L.; Jiang, Z.; Zhou, Y.; Tsymbal, E. Y.; Alexandrov, V. Magnetoelectric Effect at the Ni/HfO_2 Interface Induced by Ferroelectric Polarization. *Phys. Rev. Appl.* **2019,** *12* (2), 024044.
(17) Chen, Z.; Yang, Q.; Tao, L.; Tsymbal, E. Y. Reversal of the Magnetoelectric Effect at a Ferromagnetic Metal/Ferroelectric Interface Induced by Metal Oxidation. *npj Computational Materials* **2021,** *7* (1), 204.
(18) Dmitriyeva, A.; Mikheev, V.; Zarubin, S.; Chouprik, A.; Vinai, G.; Polewczyk, V.; Torelli, P.; Matveyev, Y.; Schlueter, C.; Karateev, I.; Yang, Q.; Chen, Z.; Tao, L.; Tsymbal, E. Y.; Zenkevich, A. Magnetoelectric Coupling at the Ni/$Hf_{0.5}Zr_{0.5}O_2$ Interface. *ACS Nano* **2021,** *15* (9), 14891-14902.
(19) Vermeulen, B.; Ciubotaru, F.; Popovici, M. I.; Swerts, J.; Couet, S.; Radu, I. P.; Stancu, A.; Temst, K.; Groeseneken, G.; Adelmann, C. Ferroelectric Control of Magnetism in Ultrathin HfO_2\Co\Pt Layers. *ACS Appl. Mater. Interf.* **2019,** *11* (37), 34385–34393.
(20) Wei, Y.; Matzen, S.; Quinteros, C. P.; Maroutian, T.; Agnus, G.; Lecoeur, P.; Noheda, B. Magneto-Ionic Control of Spin Polarization in Multiferroic Tunnel Junctions. *npj Quantum Mater.* **2019,** *4* (1), 1-6.

inplane.mp4

Video S1. In-plane projection of polarization vector of the La:HZO taking considering the different in-plane variants of the orthorhombic (111) oriented crystallites. In the video the vectors of the 4 in-plane crystalline variants with the three possible polarization orientation are plotted. Time resolved it is plotted the projection of these 12 possible in-plane polarization directions at the given angle. The angular dependence of the projected polarization is also plotted. It can be observed that the in-plane anisotropy is small, and therefore no in-plane preferential directions should exist.
